# Severe Acute Respiratory Syndrome Coronavirus 2 Antibodies Among Healthcare Workers After Vaccine Administration in an Intensive Care Unit

**DOI:** 10.7759/cureus.20579

**Published:** 2021-12-21

**Authors:** Cláudia Lemos, Sofia Ferreira, Cláudio Gouveia, Érica Mendonça, Ana Marta Mota, Mariana Rodrigues, José Alves, Susana Chaves, Graça Andrade, José J Nóbrega

**Affiliations:** 1 Intensive Care Department, Hospital Central do Funchal, Funchal, PRT; 2 Internal Medicine, Centro Hospitalar Lisboa Ocidental, Lisbon, PRT; 3 Pathology, Hospital Central do Funchal, Funchal, PRT; 4 Centro de Investigação Dra Maria Isabel Mendonça, Hospital Central do Funchal, Funchal, PRT; 5 Intensive Care Department, Hospital Central do Funchal, Serviço de Saúde da Região Autónoma da Madeira (SESARAM) EPERAM, Funchal, PRT

**Keywords:** immunoglobulin g, vaccine, healthcare workers, sars-cov-2 antibody response, covid-19

## Abstract

Severe acute respiratory syndrome coronavirus 2 (SARS-CoV-2) and coronavirus disease 2019 (COVID-19) emerged in China in December 2019. Healthcare workers (HCWs) are one of the high-risk groups of infection and knowledge of the seroprevalence of SARS-CoV-2 antibodies among this class is very important, not only to understand the spread of COVID-19 among health institutions but also to assess the success of public health interventions. The objective of this prospective study was to determine the seroprevalence of COVID-19 immunoglobulin G (IgG) antibodies after vaccine administration and assess the symptomatology associated with the number of IgG antibodies. A total of 75 HCWs from an intensive care unit were studied three and six months after the second administration of the COVID-19 vaccine. They were divided into three groups: IgG antibodies between 4,160 and 6,350 (group one), greater than 6,350 (group two), and less than 4,160 (group three). After the first administration of the vaccine, 80% had symptoms in both groups one and two, whereas only 13.8% had symptoms in group three. After the second dose of the vaccine, all elements of group one and 80% of group two developed symptoms, but only 40% of group three manifested symptoms. With the exception of one, all professionals showed a decrease in the number of IgG antibodies from three to six months. Our findings show that professionals with a higher number of IgG antibodies had more symptoms and that these rapidly declined over the three-to-six-month period.

## Introduction

Severe acute respiratory syndrome coronavirus 2 (SARS-CoV-2) has swept the globe at a shocking rate, with a reported 265,970,247 cases and 5,267,137 deaths [1]. The World Health Organization named the disease caused by SARS-CoV-2 as coronavirus disease 2019 (COVID-19) on February 11, 2020, and with the rapid spread of the virus, it was declared a global pandemic [2,3]. Healthcare workers (HCWs) working on the frontline play an important role in supporting patients infected with coronavirus and have a higher risk of infection [4]. There has been an extraordinary global effort to identify new diagnostic tests, treatments, and, more lately, vaccines against the virus and the associated disease, COVID-19 [5]. After vaccination, antibodies specific to COVID-19 are substantial for neutralization and clearance of the virus and are quantified by in vitro neutralization assays [6]. After SARS-CoV-2 exposure, antibody titers will be important biomarkers not only for the evaluation of humoral response but also their protective efficacy since there is a scarcity of data regarding post-infection immunity [7].

## Materials and methods

HCWs were informed about the conduction of the COVID-19 antibody serological study and approval was acquired from the hospital's ethics committee (IRB approval number: E.21078233) to analyze the prevalence of SARS-CoV-2 antibodies among HCWs of the ICU after Pfizer-BioNTech vaccine administration. The HCWs included doctors (n = 5), nursing staff (n = 57), operational assistants (n = 11), and administrative staff (n = 2).

The study population was divided into three groups: immunoglobulin G (IgG) antibodies between 4,160 and 6,350 (group one), greater than 6,350 (group two), and less than 4,160 (group three). Clinical and demographic data such as sex, age, symptoms after the first and second administration of the vaccine, contact with positive patients, comorbidities, and the difference in antibodies between three and six months were analyzed.

The SARS-CoV-2 IgG II Quant assay is a chemiluminescent microparticle immunoassay used for the qualitative and quantitative determination of IgG antibodies to the receptor binding domain of the S1 subunit of the spike protein of SARS-CoV-2 in human serum and plasma. If the IgG antibodies were between 4,160 and 6,350, there was a 95% probability of presence of neutralizing antibodies, and if they were superior to 6,350, there was a 99% probability.

Data analysis was performed using the chi-square test for categorical variables and the one-way analysis of variance (ANOVA) test to compare means between groups. The Wilcoxon test was used to analyze differences between antibodies. Data analysis was performed using SPSS for Windows version 25.0 (IBM SPSS Statistics, Armonk, NY).

## Results

A total of 75 HCWs were included in this study. Group one had an average age of 33 years and all were female. As for group two, the average age was 40 years, where 60% were female and 40% were male. Group three had an average age of 39 years and 61.5% were female and 38.5% were male. In group one, most HCWs did not have comorbidities (60%); however, in groups two and three, most had associated pathologies (80% and 83.1%, respectively). The most frequent were hypertension, hypothyroidism, and asthma.

There was a prevalence of 6.66% of SARS-CoV-2 antibodies in groups one and two and 86.7% in group three. After the first administration of the vaccine, 80% of HCWs had symptoms in both groups one and two and only 13.8% had symptoms in group three. After the second dose of the vaccine, all elements of group one and 80% of group two had symptomatology, but only 40% of group three developed symptoms.

Table 1 shows the percentage of symptoms in the three groups after the two doses of the COVID-19 vaccine.

**Table 1:** Symptoms in the three groups after the two doses of the COVID-19 vaccine. COVID-19, coronavirus disease 2019.

Symptoms after administration of the vaccine	Group 1	Group 2	Group 3
First dose	80%	80%	13.8%
Second dose	100%	80%	40%

Symptoms included fever, asthenia, myalgia, arthralgia, cephalgia, vomiting, diarrhea, and dizziness. The frequency of symptoms in the three groups after the first dose of the COVID-19 vaccine is shown in Table 2.

**Table 2:** Frequency of symptoms in the three groups after the first dose of the COVID-19 vaccine. COVID-19, coronavirus disease 2019.

Symptoms after administration of the vaccine	Group 1	Group 2	Group 3
Fever	70%	65%	20%
Asthenia	71%	60%	15%
Myalgia	65%	55%	15%
Arthralgia	45%	50%	10%
Cephalgia	40%	40%	10%
Vomiting	25%	30%	6%
Diarrhea	20%	30%	5%
Dizziness	5%	10%	3%

After the second administration of the vaccine, the frequency of symptoms was higher. The frequency of symptoms in the three groups after the second dose of the COVID-19 vaccine is depicted in Table 3.

**Table 3:** Frequency of symptoms in the three groups after the second dose of the COVID-19 vaccine. COVID-19, coronavirus disease 2019.

Symptoms after administration of the vaccine	Group 1	Group 2	Group 3
Fever	90%	80%	40%
Asthenia	75%	80%	20%
Myalgia	85%	85%	20%
Arthralgia	70%	60%	15%
Cephalgia	65%	65%	15%
Vomiting	65%	55%	10%
Diarrhea	40%	50%	5%
Dizziness	5%	10%	3%

Most professionals in group two had regular contact with infected patients (80%) as well as group three (63.1%); however, the majority of group one had no contact with these patients (60%).

At three months, mean and median IgG antibodies were 2668.6 and 2373.1, respectively, where the minimum was 3.4 and the maximum was 8336.8. At six months, we calculated a mean of 1373.5 and a median of 1256.9 IgG antibodies, where the minimum was 4.6 and the maximum was 5192.7.

**Figure 1:**
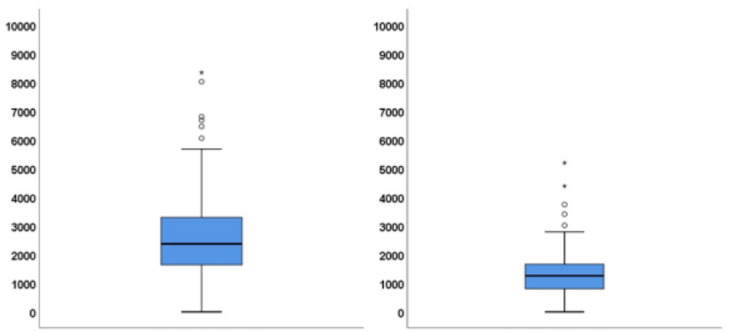
Mean and median of IgG antibodies at three months (left) and six months (right) after the second dose of vaccine administration. IgG, immunoglobulin G.

The mean and median of IgG antibodies at three and six months after the second dose of the COVID-19 vaccine are depicted in Figure 1.

With the exception of one, all HCWs showed a decrease in the number of antibodies in the three-to-six-month period after the administration of the second dose.

## Discussion

Humoral and cell-mediated response against SARS-CoV-2 is one of the major aspects for understanding viral clearance and vaccination effectiveness. One study confirmed that HCWs reach adequate humoral and cellular response after a single dose of an mRNA vaccine [8]. Since SARS-CoV-2 has adversely impacted global health with significant mortality and morbidity, a prompt diagnosis is of utmost importance [9]. Studying the kinetics of SARS-CoV-2 antibodies is beneficial not only to monitor trends in the virus transmission but also to study the impact of the COVID-19 infection in the community [10].

In the present prospective study, we found a substantial decline in IgG levels in the three-to-six-month period after the second dose of the COVID-19 vaccine, except in one HCW. Our findings are similar to those of Nag et al. [11]. Mishra et al. found a substantial decline in levels of IgG from peak values one month after the second dose and a sharper decay rate of 72% and 17% from two different COVID-19 vaccines [12]. Two studies demonstrate a significant antibody decline three and six months post-vaccination [13,14].

Studies observed that spike IgG concentrations are durable, with modest declines after six to eight months. Kinetics of humoral response over a longer time period should be evaluated to obtain information on the long-term immunity in vaccinated subjects [15].

Vaccination has been shown to provide significantly better protection against severe diseases. Boosting with vaccines is an effective strategy to combat the waning of immunity and the current variants of concern [16].

In group two, we found HCWs who presented with the highest number of IgG antibodies (superior to 6,350). This group was the one with a higher average age and female ratio, with a significant percentage of comorbidities and symptomatology after the first and second administration of the COVID-19 vaccine and all had consistent contact with infected patients. In group one, we found the professionals with antibodies between 4,160 and 6,350. They were all female, younger, mostly with no comorbidities, and had no contact with COVID-19 patients. Like group two, they predominantly had symptoms after the first and second vaccine administration. Group three comprised HCWs with the lowest number of antibodies, inferior to 4,160. This group was mostly female with associated previous pathologies and with contact with infected patients. However, they had fewer symptoms after vaccine administration.

Limitations of our clinical study include the small sample size.

## Conclusions

Our study concluded that professionals with a higher number of antibodies presented with more symptoms after vaccine administration. It also came to light that in the three-to-six-month period after vaccine administration, there was a significant decrease in the number of antibodies.

However, it is important to note that this is an area of investigation where data are still quite scarce and further studies are needed on this subject, especially about the third dose of the vaccine.
